# Hypertriglyceridaemia: contemporary management of a neglected cardiovascular risk factor

**DOI:** 10.21542/gcsp.2021.19

**Published:** 2021-10-30

**Authors:** Tina Z. Khan, Ulrike Schatz, Stefan R. Bornstein, Mahmoud Barbir

**Affiliations:** 1 Department of Cardiology, Harefield Hospital, Royal Brompton & Harefield NHS Foundation Trust Hospital, Hill End Road, Harefield UB9 6JH, United Kingdom; 2 University Hospital Carl Gustav Carus, Fetscher Street 74, Dresden 01307, Germany

## Abstract

Hypertriglyceridaemia represents one of the most prevalent lipid abnormalities, however it is often eclipsed by focus on LDL cholesterol and is frequently overlooked by clinicians, despite it being an important cardiovascular risk factor. For most patients, hypertriglyceridaemia arises from a combination of environmental factors and multiple genetic variations with small effects. Even in cases with apparent familial clustering of hypertriglyceridaemia, a monogenetic cause is rarely identified. Common secondary causes include obesity, uncontrolled diabetes, alcohol, and various commonly used drugs. Correction of these factors, along with lifestyle optimisation, should be prioritised prior to commencing medication. The goal of drug treatment is to reduce the risk of cardiovascular disease in those with moderate hypertriglyceridaemia and the risk of pancreatitis in those with severe hypertriglyceridaemia. Recent and ongoing trials demonstrate the important role of triglycerides (TG) in determining residual risk in patients with cardiovascular disease (CVD) already established on statin therapy. Novel and emerging data on omega-3 fatty acids (high-dose icosapent ethyl) and the selective PPAR modulator pemafibrate are eagerly awaited and may provide further clarity for clinicians in determining which patients will benefit from TG lowering and help inform clinical guidelines. There are numerous novel therapies on the horizon that reduce TG by decreasing the activity of proteins that inhibit lipoprotein lipase such as apolipoprotein C-III (including Volanesorsen which was recently approved in Germany) and ANGPTL 3/4 which may offer promise for the future.

## Introduction

Although hypertriglyceridaemia represents a common lipid disorder, uncertainty and debate remain regarding its prognostic significance and optimal modes of treatment. There are numerous causes for hypertriglyceridaemia and the diagnostic role of genetic testing is often unclear. Whilst the causative role of LDL-cholesterol in atherosclerotic cardiovascular disease has been long established, with an international consensus on the importance of its management, there is far less clarity on the prognostic implications and management of hypertriglyceridaemia and raised VLDL.

Hypertriglyceridaemia is an uncommon but well-established aetiology of acute pancreatitis, although the exact mechanism by which hypertriglyceridaemia causes pancreatitis is not yet clearly understood, although it may involve metabolism of excessive triglycerides by pancreatic lipase to free fatty acids (FFA), leading to pancreatic cell injury and ischaemia^1^.

Dyslipidaemia, characterized by hypertriglyceridaemia and increased LDL cholesterol, is also commonly associated with non-alcoholic fatty liver disease (NAFLD) and in turn, cardiovascular disease (CVD) is the most common cause of mortality in NAFLD patients. Alterations in hepatic lipid and lipoprotein metabolism are major contributors towards the increased CVD risk in NAFLD patients^2^.

This article aims to explore these questions and review contemporary management options for treating hypertriglyceridaemia.

### Definitions

Hypertriglyceridaemia is commonly defined as fasting serum triglycerides of 1.7 mmol/L (150 mg/dL) or above^3^. However, there is international disparity in terms of defining the threshold of severe hypertriglyceridaemia. The European Atherosclerosis Society/European Society of Cardiology (ESC/EAS) classify severe hypertriglyceridaemia as concentrations of at least 10 mmol/L (880 mg/dL), given pancreatitis is rarely seen below this threshold^4^.

In contrast, the American Heart Association/American College of Cardiology (AHA/ACC) guidelines on lipid treatment consider serum triglycerides of ≥5.6 mmol/L (500 mg/dL) or above as severe hypertriglyceridaemia, indicative of risk for pancreatitis^5^.

**Figure 1.**
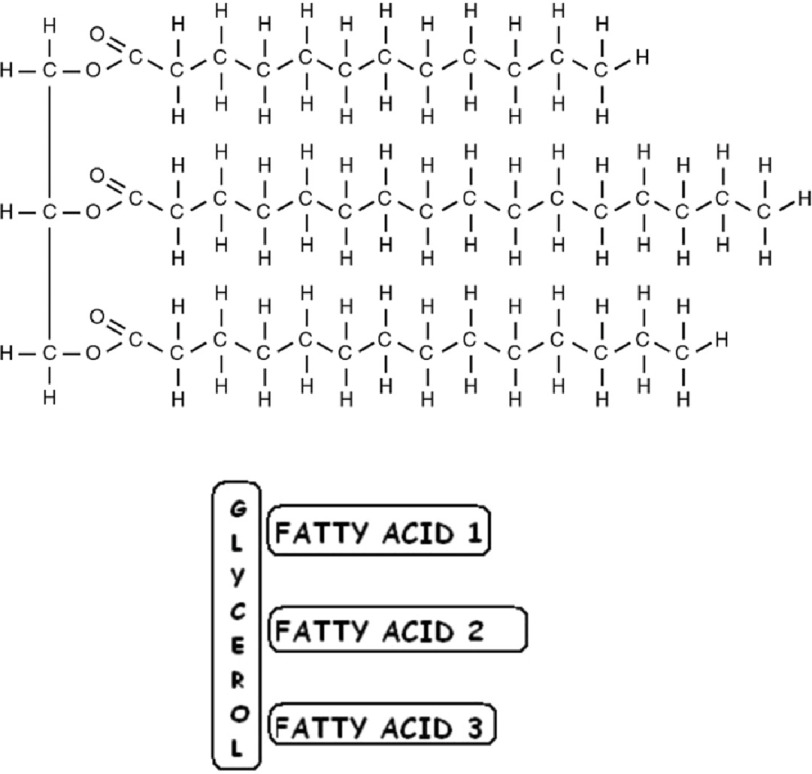
The biochemical structure of triglycerides (actual structure shown above a simplified version). Upper image: Illustration from Anatomy & Physiology, Connexions Web site. http://cnx.org/content/col11496/1.6/, Jun 19, 2013. Lower image: *Diagram of triglyceride structure by Ruth Lawson, Otago Polytechnic. Both images *via* WikiMedia*.

The biochemical structure of triglycerides which consists of a glycerol backbone with three fatty acid chains attached is shown in Figure 1 below.

### Epidemiology

Hypertriglyceridaemia is abundantly prevalent worldwide. Based on analysis of nine European population cohorts, the prevalence of hypertriglyceridaemia (serum triglycerides > 1.7 mmol/L) was 36.4% in men and 24.8% in women^6^. Similarly, the prevalence of hypertriglyceridaemia amongst US adults was 33.3% in 2001–04^7^ and subsequently fell to 25.9% in 2007–14^8^, suggesting an encouraging epidemiological trend.

In a large epidemiological study that aimed to identify ethnic differences in dyslipidaemia compared with non-Hispanic whites, every minority subgroup including Asian Americans (Asian Indian, Chinese, Filipino, Japanese, Korean, or Vietnamese) and Mexican Americans, had an increased prevalence of high triglycerides, with the exception of Blacks^9^.

### Causes of hypertriglyceridaemia

Most patients with hypertriglyceridaemia do not have a detectable genetic cause, and the elevated triglycerides are likely to arise from a combination of multiple genetic variations with cumulative effects and environmental influences^3^. Even in cases with apparent familial clustering of hypertriglyceridaemia, a monogenetic cause is rarely identified.

### Secondary causes of hypertriglyceridaemia

**Figure 2.**
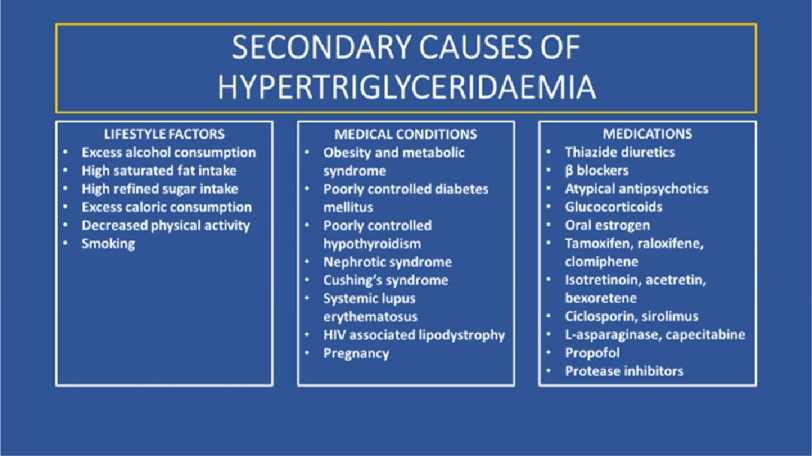
Summary of secondary causes of hypertriglyceridaemia.

Secondary causes of hypertriglyceridaemia are much more prevalent than primary causes. Lifestyle risk factors include excess alcohol consumption; a sub-optimal diet including high saturated fat intake, high refined sugar intake, excess caloric consumption; decreased physical activity and smoking^3^. Numerous medical conditions or physiological states also cause predisposition to hypertriglyceridaemia including; obesity and metabolic syndrome, poorly controlled diabetes mellitus and hypothyroidism, nephrotic syndrome, Cushing’s syndrome, systemic lupus erythematosus (SLE), HIV-associated lipodystrophy, and pregnancy^3^. In addition, hypertriglyceridaemia may be iatrogenic and secondary to medications including; thiazide diuretics, non-selective β-blockers, atypical antipsychotics, glucocorticoids and oral estrogen^3^. Secondary causes of hypertriglyceridaemia are summarised in Figure 2.

### Primary hypertriglyceridaemia

Primary hypertriglyceridaemia, on the other hand, is rarer than secondary hypertriglyceridaemia. Frequently, secondary factors unmask an underlying, often polygenic, primary hypertriglyceridaemia.

Familial chylomicronemia syndrome (FCS) is an exceedingly rare autosomal recessive disorder with an estimated prevalence of only one in a million. Bi-allelic mutations in lipoprotein lipase account for most cases^10,11^, followed by mutations in apolipoprotein A5^12^, believed to stabilise the dimeric structure of lipoprotein lipase. Affected individuals usually have recurrent pancreatitis from childhood, associated with increased morbidity and mortality, and may show eruptive xanthoma and lipaemia retinalis when serum triglyceride concentrations are above 50 mmol/L^3^.

It is now recognised that chylomicronemia can be either monogenic–as in the case of FCS–or it can be multifactorial chylomicronemia (MCM), which is a polygenic disorder. A recent comparative study sought to identify the characteristic differences between patients with FCS *versus* MCM^13^. This study demonstrated that FCS patients presented with a significantly higher frequency of pancreatitis (60% *vs.* 6%), multiple pancreatitis (48% *vs.* 3%), abdominal pain (63% *vs.* 19%) and a lower frequency of metabolic disturbances than in the MCM group (*p* < 0.0001)^13^. In addition, the frequency of cardiovascular events was higher in the MCM group than in the FCS group (17% *vs.* 0%), although this difference was not statistically significant (*p* = 0.07).

A recent study also showed that MCM patients with a rare heterozygote variant of LPL appear to have an intermediate risk of developing pancreatitis with an intermediate phenotype between MCM patients without rare variants and FCS patients; potentially enabling identification of higher-risk MCS patients who would benefit from additional treatment^14^.

Familial hypertriglyceridaemia is a relatively common lipid disorder with moderate elevations in serum triglycerides (5–25 mmol/L) due to increased secretion of triglyceride-rich VLDL particles. Familial clustering is observed, although no genetic cause has thus far been identified^3^. It is often referred to as “benign hypertriglyceridaemia”, given that it is not independently associated with increased risk for CVD in the absence of other cardiovascular risk factors^15^.

Familial combined hyperlipidemia is another reasonably common disorder in which affected individuals may have elevated cholesterol, triglycerides, or both, with familial clustering amongst first degree relatives, although despite the strong familial predilection, a monogenetic basis of this disorder has not been identified^3^. A strong predisposition to premature ASCVD and characteristically elevated apolipoprotein B concentrations (> 90th percentile or 120 mg/dL) helps to differentiate this condition from familial hypertriglyceridaemia^16^.

Familial (type III) dysbetalipoproteinemia is a rare condition strongly associated with premature cardiovascular disease, which involves a genetic predisposition combined with environmental factors causing its manifestation^17^. Hepatic clearance of chylomicron remnants and VLDL remnants requires apolipoprotein E, which is slowed in the presence of the E2/E2 phenotype. However, given there are alternative pathways which help in remnant clearance^18^ most individuals with the E2/E2 phenotype will not necessarily exhibit significant dyslipidaemia. In cases where secondary factors increase the generation of triglycerides (*e.g.*, obesity, excess calorie intake, excess alcohol consumption, estrogen) or decrease their clearance (*e.g.*, hypothyroidism); the alternate pathways become overwhelmed, causing accumulation of remnants^3^.

### Current guidelines

Guidelines for the management of hypertriglyceridaemia have been issued from numerous international authorities including the ESC/EAS^4^, AHA/ACC^5^, and the Endocrine Society^19^. The unifying feature common to all three is the strong emphasis on the correction of secondary causes of hypertriglyceridaemia. The role of lifestyle interventions is emphasised, with prioritisation of weight reduction and minimisation of alcohol consumption endorsed by the ESC/EAS^4^.

The threshold triglyceride concentration to initiate drug treatment to reduce the risk of pancreatitis differs: ≥5.6 mmol/L (500 mg/dL) in the AHA/ACC guidelines, 10 mmol/L (880 mg/dL) in the ESC/EAS guidelines, and 11.3 mmol/L (1000 mg/dL) in the Endocrine Society guidelines. All three societies recommend fibrate (preferably fenofibrate) and omega-3 fatty acids, with additional inclusion of niacin in the ESC/EAS guidelines to reduce severe hypertriglyceridaemia and risk of pancreatitis^3^. Statin therapy is also unanimously recommended by all three guidelines for ASCVD risk reduction, especially in those with moderate hypertriglyceridaemia^3^.

**Figure 3.**
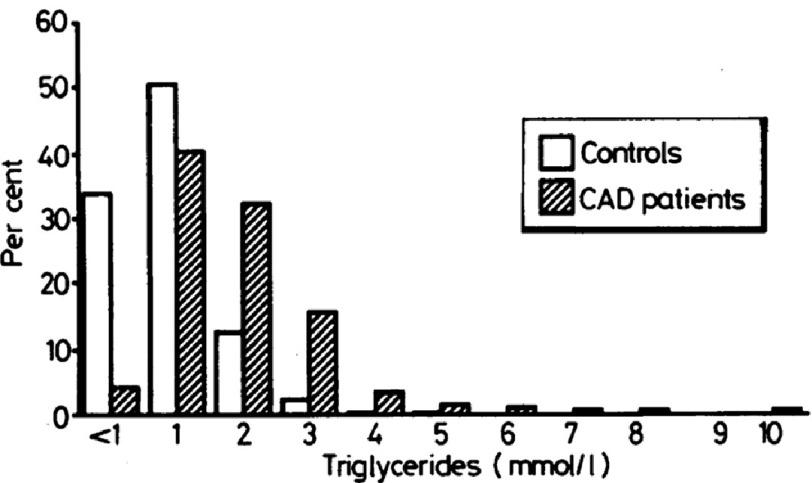
Higher TG levels in patients with CAD *versus* healthy controls (reproduced with permission)^20^.

Ultimately the rationale for treating hypertriglyceridaemia needs to be guided by a strong evidence base confirming the role of raised triglycerides as a cardiovascular risk factor. Barbir et al. performed a case-control study comparing lipid parameters including triglyceride levels in patients with angiographically confirmed coronary artery disease (CAD) *versus* healthy controls and demonstrated that triglyceride levels (mmol/L) were significantly higher in the presence of CAD with a mean value of 2.43 (SD 1.37) compared to 1.40 (SD 0.71) in controls (see Figure 3)^20^. In addition, hypertriglyceridaemia was noted in 52% of the patients with coronary artery disease in contrast to 29% of the controls^20^.

More recently, moderate-to-severe hypertriglyceridaemia was found to be associated with a significantly increased risk of all-cause mortality and ASCVD events in a large cohort of low-to-moderate cardiovascular risk individuals in a real-world clinical setting^21^. A large European epidemiological study also demonstrated an association between hypertriglyceridaemia and aortic valve stenosis^22^. However debate remains over the role of hypertriglyceridaemia as an independent cardiovascular risk factor. It is not triglycerides themselves which are atherogenic, but the remnant cholesterol of TG rich lipoproteins, which contain ApoB. In severe hypertriglyceridaemia, cardiovascular risk is not further increased as chylomicrons are too large to enter vessel walls.

In the Emerging Risk Factors Collaboration Study, involving more than 300,000 participants from 68 prospective studies, raised fasting and non-fasting serum triglycerides were associated with increased risk of coronary heart disease (CHD), but not after adjustment for non-HDL cholesterol.^23^

### Management

#### Lifestyle

A key aspect of treating hypertriglyceridaemia is to manage lifestyle factors associated with elevated TG, which is often overlooked by busy clinicians keen to initiate medication in the first instance.

##### Weight loss

Serum triglycerides are significantly more responsive than serum cholesterol to weight changes. Even a modest 5% weight loss *via* caloric restriction alone has been demonstrated to significantly reduce serum triglycerides by approximately 10%, despite minimal change in other lipid parameters^24^. A 5–10% weight loss target is reasonable in overweight patients with hypertriglyceridaemia, which would typically reduce serum triglycerides by approximately 20%^3^.

##### Dietary changes

Most studies suggest that the optimal diet for patients with hypertriglyceridaemia should promote weight loss, comprise of no more than 50–60% carbohydrate sources, with mainly fibre-rich complex carbohydrates (such as whole grain and fruits and vegetables) and should avoid fructose^3^. Saturated fat should be restricted to below 7% of total energy intake, and increased consumption of monounsaturated fatty acids (*e.g.*, nuts, olive oil) and marine omega-3 polyunsaturated fatty acids (oily fish) is recommended. Above all, no alcohol consumption is advised^3^. However, these general recommendations require modification in patients with extreme hypertriglyceridaemia due to familial chylomicronemia syndrome, in whom restriction of total dietary fat to below 10–15% of total energy intake (15–20 g/day) is required^25^.

##### Exercise

A recent small, randomised trial demonstrated that 45 min of aerobic exercise five days a week significantly reduced fasting serum triglycerides^26^, indicating that recommending at least 45 min of moderate intensity exercise, five days per week in those who are able is likely to be beneficial.

##### Alcohol

In people with normal triglyceride concentrations, moderate alcohol consumption has modest effects on serum triglycerides, however chronic alcohol misuse leads to significant elevation^27,28^. In patients with primary hypertriglyceridaemia, even moderate alcohol consumption has a significant effect on triglycerides and excessive alcohol can severely exacerbate hypertriglyceridaemia. For these reasons complete abstinence is strongly recommended for such people.

#### Medication

All commonly available lipid-lowering drugs such as statins, ezetimibe, PCSK9 inhibitors, fibrates, omega-3-fatty acids, and niacin affect TG levels. LDL-lowering drugs such as statins, ezetimibe and PCSK9 inhibitors usually have a modest TG lowering effect (5–15%), whilst fibrates, omega-3-fatty acids, and niacin have more profound effects (25–45%)^29^.

##### Statins

Although no statin trials have been done exclusively in hypertriglyceridemic patients, subgroup analyses of major statin trials have demonstrated a similar or greater benefit compared with patients with normal triglyceride levels^30–32^. Given the abundance of evidence which has established the efficacy of statins for both primary and secondary prevention of cardiovascular disease^33^, initiation of statin therapy in all patients with hypertriglyceridaemia and elevated risk of ASCVD seems an advisable strategy.

##### Fibrates

Fibrates can reduce TG by up to 70%, although with marked variation between individuals^29^. Several large placebo-controlled trials have shown cardiovascular benefit for both primary and secondary prevention^34,35^, although no mortality benefit was seen. A prominent ongoing ASCVD outcome trial using the new agent, pemafibrate–a selective PPAR modulator, is likely to provide further insight^36^. In clinical practice, many specialists consider fibrate therapy in patients with elevated ASCVD risk and persistent hypertriglyceridaemia despite reaching the target LDL-C and lifestyle modifications.

##### Omega-3 fatty acids

Marine long chain omega-3 polyunsaturated fatty acids, docosahexaenoic acid, and eicosapentaenoic acid can lower serum triglycerides by 30–50%^37^ by decreasing VLDL synthesis *via* numerous mechanisms^38^ and by enhancing lipoprotein lipase mediated clearance of triglycerides^39^.

A recent large scale study, REDUCE-IT, evaluated the effect of 4 g icosapent ethyl (Vascepa) in > 9,000 high-risk patients with elevated TG levels on background statin therapy and demonstrated a dramatic risk reduction (HR 0.75) for ASCVD events^40^. Uncertainties remain regarding whether the observed benefit relates to the particular omega-3 fatty acid formulation used (icosapent ethyl), the high daily dose of 4 g, the carefully selected study population, or potentially deleterious effect of the mineral oil comparator used as placebo^29^. Furthermore, the strongly positive outcomes appeared to be mediated by factors other than isolated TG reduction, since the observed benefit was independent of the baseline TG level^29^. There is another large ongoing study using high-dose omega-3 fatty acids which will hopefully provide more clarity^41^.

In a randomised study, the efficacy and safety of docosahexaenoic acid (10 g/day) and bezafibrate (400 mg/day) for 3 months were compared in 87 cardiac transplant recipients with serum total cholesterol > 6.5 or triglycerides > 2.8 mmol/liter, or both^42^. Although docosahexaenoic acid had no significant effect on other lipid parameters, it was as effective as bezafibrate in reducing triglycerides (36% and 31%, respectively). In addition, bezafibrate significantly increased serum creatinine indicating a potentially adverse effect on renal function^42^.

##### Niacin

Despite the favourable lipid effects of niacin–which reduces serum triglycerides and increases HDL cholesterol, as well as reducing LDL cholesterol–two large clinical trials did not demonstrate any prognostic benefit^43,44^. Niacin therefore has a limited role for additional risk reduction in patients with well controlled lipids on statin therapy.

### Lipoprotein apheresis

In the absence of other lipid abnormalities such as significantly raised LDL cholesterol and raised Lipoprotein(a) [Lp(a)]; isolated hypertriglyceridaemia has not been established as an indication criteria for lipoprotein apheresis, which involves the extra-corporeal removal of lipoproteins from the blood. This is due to the fact that TG levels may decrease by 50% with lipoprotein apheresis, however plasma triglyceride levels rebound to baseline levels within 24 hours^45^ compared to a greater magnitude of lipid lowering observed for LDL and Lp(a), with a slower rebound effect^45,46^.

### Emerging novel therapies

There are numerous promising novel therapies on the horizon that offer future hope for the management of hypertriglyceridaemia. Most of these reduce TG by increasing lipoprotein lipase mediated clearance of TG by decreasing the activity of proteins that inhibit lipoprotein lipase such as apolipoprotein C-III and ANGPTL 3/4^3^.

Volanesorsen is an antisense oligonucleotide that inhibits apolipoprotein C-III (ASO Apo CIII) and has been shown in phase II trials to reduce serum triglycerides by 31–71% in a dose dependent manner^47^. This drug also reduced serum triglycerides by 56–86% in patients with familial chylomicronemia syndrome who are deficient in lipoprotein lipase^48^, indicating that it also aids clearance of TG through lipoprotein lipase-independent pathways. Volanesorsen was recently approved in Germany for use in familial chylomicronemia syndrome. Outcome data from phase III trials are eagerly awaited to provide data on the prognostic impact of this promising therapy.

Both a monoclonal antibody (Evinacumab) and an antisense oligonucleotide to ANGPTL3 have also been developed and both show promise following initial dose-finding studies^49,50^, with further clinical trials awaited.

Gene therapy for lipoprotein lipase deficiency delivered through an adeno-associated viral vector, alipogene tiparvovec, has also been shown to be effective in patients with familial chylomicronemia syndrome for reducing triglyceride levels within 12 weeks^51^, although unfortunately in most cases, TG drifted back to baseline levels within 6 months.

**Figure 4.**
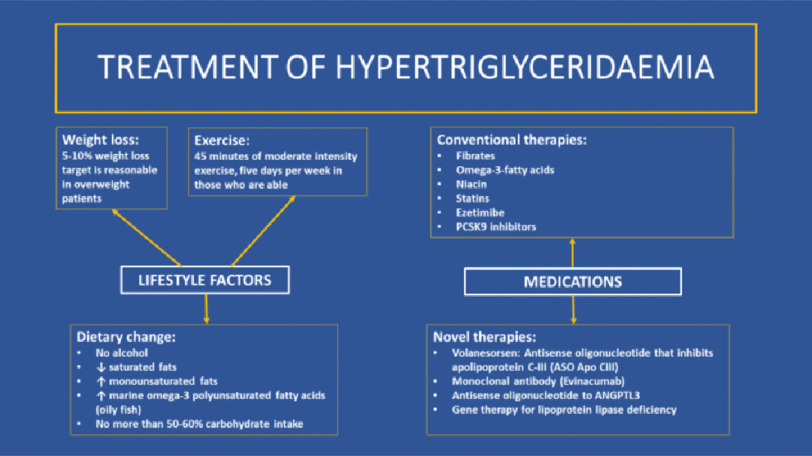
Treatment of hypertriglyceridaemia.

Lifestyle strategies and medication options for the treatment of hypertriglyceridaemia are summarised in Figure 4.

## Conclusions

Hypertriglyceridaemia represents one of the most prevalent lipid abnormalities with moderate hypertriglyceridaemia predisposing to CVD, and severe hypertriglyceridaemia (≥10 mmol/L) predisposing to acute pancreatitis. Hypertriglyceridaemia is often eclipsed by the spotlight on LDL cholesterol and deserves more attention from clinicians as an important cardiovascular risk factor. In most cases, triglyceride elevations arise from a combination of environmental factors and multiple genetic variations with small effects. Genetic testing in patients with hypertriglyceridaemia is generally not recommended unless FCS is strongly suspected^52^.

Common secondary causes include obesity, uncontrolled diabetes, alcohol, and various commonly used drugs. Correcting these factors and lifestyle optimisation including dietary modification should be prioritised prior to commencing medication. The goal of drug treatment is to reduce the risk of cardiovascular disease in those with moderate hypertriglyceridaemia and the risk of pancreatitis in those with severe hypertriglyceridaemia.

Older trials involving fibrates and niacin did not provide conclusive evidence of ASCVD risk reduction. However, recent and ongoing studies demonstrate the important role of TG as a determinant of residual risk in patients with CVD established on statin therapy. Recent data on omega-3 fatty acids (high-dose icosapent ethyl) and the selective PPAR modulator pemafibrate has been promising and further emerging data may provide more clarity and identify patients who will benefit from TG lowering. Numerous novel therapies on the horizon that reduce TG by decreasing the activity of proteins that inhibit lipoprotein lipase such as apolipoprotein C-III and ANGPTL 3/4, including Volanesorsen (ASO Apo CIII) approved in Germany for FCS with high risk of pancreatitis, offer promise for the future.
